# Automated Software Evaluation in Screening Mammography: A Scoping Review of Image Quality and Technique Assessment

**DOI:** 10.3390/curroncol32100571

**Published:** 2025-10-15

**Authors:** Kelly M. Spuur, Clare L. Singh, Dana Al Mousa, Minh T. Chau

**Affiliations:** Faculty of Science and Health, Charles Sturt University, Wagga Wagga, NSW 2678, Australia; csingh@csu.edu.au (C.L.S.); dalmousa@csu.edu.au (D.A.M.); schau@csu.edu.au (M.T.C.)

**Keywords:** mammography, breast screening, automated software, PGMI, compression, breast cancer, image quality

## Abstract

**Simple Summary:**

Breast cancer screening with mammography saves lives by detecting cancers early, but the quality of the images is critical for accurate diagnosis. Traditionally, radiographers judged image quality by eye, but this method can be inconsistent and subjective. New software now provides automated feedback on breast positioning, image clarity, and compression, helping radiographers improve their technique and produce higher quality images. This review looked at the current research on these tools and found they can reduce repeat imaging, support radiographer training, and make screening more consistent. However, the studies so far have only been undertaken in high-resource settings, and none have shown whether the software directly improves cancer detection. These findings suggest that automated evaluation has strong potential to improve breast screening services, but more research is needed to confirm its impact on patient outcomes and to guide wider use in different healthcare settings.

**Abstract:**

Background: Standardised breast positioning and optimal compression are critical components of effective breast cancer screening. This scoping review aims to report the current landscape of automated software tools developed for image quality assessment and mammographic technique evaluation, and to examine their reported impact. Methods: A scoping review was undertaken across PubMed (MEDLINE), Scopus, and Emcare. Eligible studies were published between January 2014 and March 2025 and investigated the use of automated software or artificial intelligence-based tools to assess image quality, breast positioning, or compression in mammography or digital breast tomosynthesis. Results: Automated software was predominantly utilised in high-resource settings, where it provided benchmarked feedback, reduced the subjectivity inherent in traditional visual grading systems, and supported radiographer learning and skill development with measurable improvements. However, radiographer training in these systems, the impact of software on clinical workflow, and barriers to implementation, particularly in low-resource settings, were insufficiently addressed in the literature. Furthermore, no studies reported on the relationship between software-generated metrics and breast cancer screening outcomes. Conclusions: Automated software for image quality evaluation represents a significant advancement in breast screening, illustrating the potential of technology to strengthen the screening-to-treatment continuum in breast cancer care. Nonetheless, widespread adoption requires evidence that these tools directly contribute to improved cancer detection outcomes to justify their uptake.

## 1. Introduction

Breast cancer remains a critical global health challenge, being the most prevalent cancer in women and a leading cause of cancer-related mortality worldwide [1]. In 2022 an estimated 2.3 million women were diagnosed with breast cancer and 685 000 died from the disease. Future projections exceed 3 million new cases and 1 million deaths per year by 2040 [1,2]. Mammography is the gold standard imaging modality to detect breast cancer and has two types of acquisition, conventional 2D mammography and digital breast tomosynthesis (DBT) [3]. Mammography is typically accessible through two distinct settings: screening and diagnostic. Where organized mammography screening programs have been implemented as a proactive response to breast cancer incidence, these programs have demonstrably reduced mortality through pre-clinical detection [4,5,6]. Countries with regular mammographic screening report significantly fewer breast cancer deaths (approximately 3.7 fewer per 100,000 women overall, and over 10 fewer per 100,000 in the 50–74 age group) compared to countries without systematic screening [4,5,6]. Mammography thus stands as the cornerstone of early breast cancer detection in global screening efforts, leveraging technology to improve outcomes across the continuum from screening to treatment.

A high standard of image quality in mammography is fundamental for maximising the detection of breast pathology including breast cancer [7,8]. There is a plethora of publications concerning issues in mammographic positioning [7,9,10,11,12,13,14,15] and image quality [15,16,17,18,19,20,21]. Key technical factors, including appropriate breast positioning, the inclusion of as much breast tissue as possible and optimal compression, directly influence the diagnostic accuracy of a mammogram [22,23]. Optimal positioning of the breast ensures the maximum amount of tissue is imaged; if any area is excluded or obscured, a cancer in that region could go undetected [7]. Indeed, poor positioning has been identified as a leading cause of image deficiencies, accounting for most American College of Radiology (ACR) accreditation failures and a significant portion of repeat examinations due to technical recall [24]. Similarly, optimal compression of the breast during imaging is crucial for image clarity as it reduces motion blur, spreads out overlapping tissues reducing superimposition, and decreases breast thickness, thereby improving contrast and tumour conspicuity [25,26]. Optimal compression also lowers the radiation dose per exam, whereas inadequate (or excessive) compression can impair lesion detectability and has been associated with a higher likelihood of interval cancers in screening cohorts and a barrier to repeat screening for some women [27,28,29].

Studies have shown that when mammographic images fail to meet accepted positioning or quality criteria, the sensitivity of cancer detection can fall from roughly 84% to about 66%, with more cancers being missed until they present clinically [16]. This highlights that the benefits of screening are tightly interwoven with the quality of the images obtained. Historically, the assessment of the image quality of the two routine mammography views, the craniocaudal (CC) and mediolateral oblique (MLO), has relied on radiographers’ visual inspection using image evaluation criteria some of which have associated scoring systems, including the Perfect (P), Good (G), Moderate (M) and Inadequate (I) (PGMI) image evaluation system (IES) [30].

**Figure 1:**
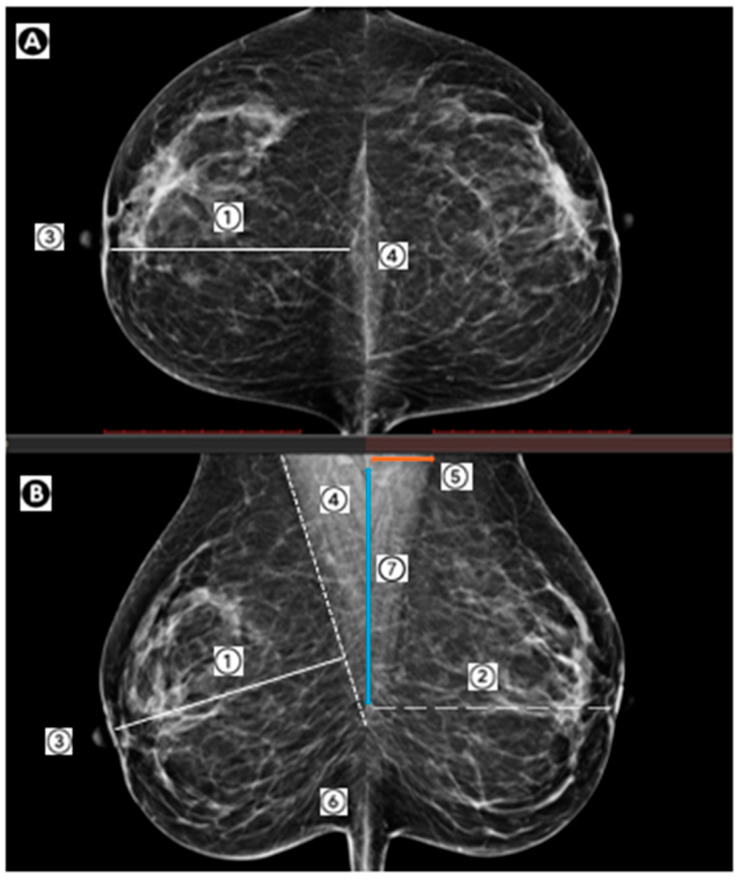
Key image quality criteria for (**A**): the craniocaudal view (CC)and (**B**): the mediolateral oblique view (MLO)1. Posterior nipple line (PNL), 2. Nipple line (NL); 3. Nipple; 4. Pectoralis major muscle; 5. Pectoral muscle width (orange); 6. Inframammary angle (IMA); 7. Pectoral muscle length (blue).

The PGMI IES was developed by United Kingdom (UK) Mammography Trainers Group with the support of the College of Radiographers in 1994 to monitor image quality in the National Health Service Breast Screening Programme (NHSBSP) [30]. The PGMI IES has been adopted by screening programs worldwide including Europe, Norway, Australia and New Zealand to support the provision of quality screening service [30,31,32,33,34]. The PGMI IES criteria assesses the inclusion of breast tissue including CC: medial border well demonstrated, nipple in profile or transected by skin edge, nipple in mid line, posterior nipple line (PNL) within 1 cm of PNL on MLO view and MLO: pectoral muscle shadow to nipple level/nipple line (NL), full width of pectoral muscle, nipple in profile or transected by skin edge, inframammary angle well demonstrated and PNL within 1 cm of PNL on MLO view(Figure 1) [30]. Additional requirements: correct image identification, and/or date of birth, correct exposure, for modality, good compression, absence of movement, correct image processing, absence of artefacts, no skin folds and symmetrical images [30]. Assessment is undertaken prospectively by radiographers and retrospectively as a form of performance review with a grade of P, G, M or I being assigned to each routine four view screening series [30].

However, such human-led image evaluation methods come with significant limitations. Visual quality grading is inherently subjective and prone to inter- and intra-observer variability. Empirical studies have found that agreement among different reviewers classifying the same mammograms can be worryingly low (with kappa values only in the 0.02–0.40 range, indicating slight to fair agreement) [35]. In one analysis, nearly half (up to 49.7%) of screening mammograms were noted to not fully satisfy all the stipulated quality criteria, suggesting either that the quality criteria are not fit for purpose or suboptimal imaging may be occurring despite visual quality assurance (QA) processes [35]. Earlier comparative research similarly concluded that both the PGMI and a newly developed Excellent (E), Acceptable (A) or Repeatable (R) (EAR) IES have poor reliability and validity for assessing mammogram quality (kappa often near 0), and that the EAR three-point scale in particular was not an adequate substitute for the four-category PGMI [20]. These findings illustrate that current non-evidence-based manual image assessment protocols, while well-intentioned, may fail to consistently identify all technical deficiencies. Additionally, the subjectivity and inconsistency in scoring can lead to frustration or false confidence among technologists, and may result in unnecessary repeat imaging (which increases patient anxiety and radiation exposure) if one evaluator deems an image “inadequate” while another might not.

Recognizing these shortcomings, some national programs have begun moving away from simplistic scoring systems. For example, the NHSBSP now mandates the use of a mammographic image assessment tool which examines the core image quality criteria of the PGMI IES without assigning a grade [36]. The NHSBSP explicitly considers the PGMI and EAR methodology “*no longer acceptable*” for routine quality review [36]. The push is toward more objective, reproducible measures of image quality that can better support radiographer performance and maintain high standards across screening services.

The arrival of automated image evaluation software represents a significant technological innovation in the radiographer facing screening phase of breast cancer care. Whilst computer aided detection software (CAD) have been used in mammography for the detection and diagnosis of breast cancer and other pathology for over twenty five years, image quality evaluation software remains relatively new [37]. Advances in software development enabling digital identification of key image evaluation criteria such as the nipple and PNL [38,39,40]. There is a clear and urgent need to systematically review the landscape of automated image quality assessment in mammography, given both the critical role of image quality in screening success and the nascency of technological solutions in this space. While mammography screening has well-established mortality benefits, one of the “*serious threats to the legitimacy*” and effectiveness of screening programs lies in quality inconsistencies and the lack of universally available automated tools for comprehensive evaluation of image quality and benchmarking [3]. No overarching review has yet been published to report and map out the utilisation of the available automated image evaluation software, what aspects of image quality or technique they address, and how extensively they have been tested or implemented globally. This is the first scoping review to systematically investigate automated software used at the image acquisition stage in radiographer-led workflows.

## 2. Materials and Methods

This scoping review followed the five-stage methodological framework outlined by Arksey and O’Malley and enhanced by Levac et al., incorporating guidance from the PRISMA-ScR (Preferred Reporting Items for Systematic Reviews and Meta-Analyses extension for Scoping Reviews) checklist [41,42]. Independent screening and reviewing of eligible studies adhered to the Preferred Reporting Items for Systematic Reviews and Meta-Analyses extension for Scoping Reviews (PRISMA-ScR) guidelines [43]. Open Science Framework (OSF) reference: DOI 10.17605/OSF.IO/7VDHW, 21 August 2025.

### 2.1. Stage 1—Identifying the Research Question

The primary research question was: What is the current landscape of automated software tools for image quality and mammographic technique evaluation in breast cancer screening, particularly in radiographer-led acquisition?

### 2.2. Stage 2—Identifying Relevant Studies

Three databases were searched: PubMed (MEDLINE), Scopus, and Emcare. The search was conducted in May 2025, and limited to studies published in the previous 10 years. Search terms combined subject headings and free-text keywords to capture three core concepts:

1. Mammography or tomosynthesis
2. Automated software/image quality/positioning/compression assessment
3. Radiographers or technologists (excluding radiologists)

The search strategy combined three main concepts using Boolean logic: (1) terms related to mammography and tomosynthesis (e.g., “mammography”, “tomosynthesis”, “breast tomosynthesis”), AND (2) terms related to image quality and technique evaluation (e.g., “image quality assessment”, “positioning assessment”, “compression analysis”, “automated evaluation”, “software evaluation”), AND (3) terms identifying radiographer involvement (e.g., “radiologic technologists”, “radiographer*”, “mammography technologist*”), while excluding studies that mentioned radiologist involvement (Table 1). The search was limited to studies published between May 2015 and May 2025.

Search strategies were adapted for Scopus and Emcare using their respective indexing terms and field tags (Table 1). Grey literature, conference abstracts, and unpublished manuscripts were excluded.

### 2.3. Stage 3: Study Selection

We included peer-reviewed studies published between May 2015 and May 2025 that examined the use of automated software or artificial intelligence tools to assess image quality, breast positioning, or compression during mammography or DBT. Studies were eligible if they:

- Were conducted in the screening setting.
- Focused on radiographer-led or technologist-applied image acquisition.
- Involved technical assessment (e.g., PGMI, EAR, or other image quality criteria/indicators).
- Included experimental, observational, technical evaluation, or quality improvement designs.
- Were published in English.

We excluded studies that:

- Conducted in the diagnostic setting (not screening).
- Focused primarily on diagnostic image interpretation or cancer detection (e.g., computor aided detection (CAD) systems).
- Involved radiologist-only workflows.
- Did not involve automated or semi-automated evaluation processes.
- Focused on other modalities (e.g., ultrasound (US), magnetic resonance imaging (MRI)).

#### Screening Process

All retrieved citations were imported into EndNote for deduplication, and then into Covidence^TM^ for screening. Two reviewers (K.M.S. and M.T.C.), independently screened titles and abstracts for relevance. Full-text articles were then reviewed against the inclusion criteria. Disagreements were resolved through discussion or consultation with a third reviewer (C.L.S.). This review followed an a priori protocol, which was agreed upon before the search and screening processes began, in line with Arksey and O’Malley’s framework. Inter-rater reliability was calculated within Covidence. The proportionate agreement between the two reviewers during title and abstract screening was 0.97826, and Cohen’s kappa was 0.71683, indicating substantial agreement.

### 2.4. Stage 4: Data Charting

A data extraction form was developed tailored to the review objectives. The form captures:

- Study characteristics (year, country, study design)
- Imaging modality (mammography, DBT)
- Type of automated software used
- Image quality metrics assessed (e.g., positioning, compression force, PGMI classification)
- Outcomes (e.g., radiographer performance, accuracy, implementation effects)
- Radiographer involvement and role
- Validation methods and comparative benchmarks (e.g., expert review, manual PGMI)

### 2.5. Stage 5: Collating, Summarising and Reporting the Results

Extracted data were reported descriptively and organized into key themes, including:

- Types of automated tools and their functions
- Methodological approaches to evaluation
- Reported benefits, limitations, and challenges
- Settings of use (e.g., clinical, educational, or audit environments)

## 3. Results

The systematic search across PubMed, Scopus, and Emcare yielded a total of 268 records (Table 1).

**Figure 2:**
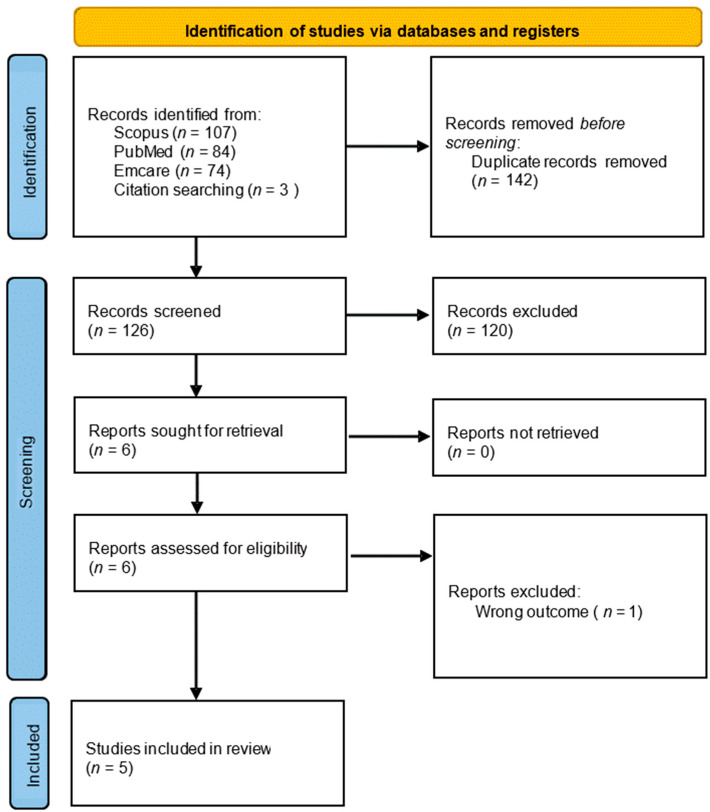
PRISMA flow diagram summarizing the identification, screening, and inclusion process.

After removing 142 duplicates, 126 studies remained for title and abstract screening. Of these, 120 were excluded based on relevance to the inclusion criteria, leaving six full-text articles for assessment. Following full-text review, one study was excluded due to wrong outcome. A final total of five studies met all inclusion criteria and were included in the review. Figure 2 presents the PRISMA flow diagram summarizing the identification, screening, and inclusion process.

Table 2 reports the key characteristics and findings of studies that evaluated automated software tools used in breast imaging. Each study investigated a specific application of automated assessment, focusing on image quality, positioning, compression or technical recall, with the majority assessing the impact on radiographer performance and workflow outcomes.

All five studies investigated the application of Volpara Analytics and/or TruPGMI^TM^ in clinical or screening settings. The studies varied in design and sample size, but all reported on radiographer-led image acquisition and software-based evaluation.

Gennaro et al. (2023) investigated the effect of automated image evaluation software on radiographer performance [44]. The study involved six breast radiographers and compared image quality outcomes before and after implementation of software tools. The authors reported increases in P and G ratings for both CC and MLO views, ranging from seven to 16 percent, as well as improvements in compression technique [44]. Eby et al. (2023) assessed the impact of software on technical repeat and recall rates across nine mammography facilities. The study found a 78 percent reduction in technical repeats and recalls following implementation. Increases were also observed in the proportion of images rated P or G, target compression pressure achieved, and overall image quality scores [45]. Pickard et al. (2022) compared manual/non automated PGMI assessments with automated evaluations using TruPGMI^TM^ [46] High agreement was observed between software and human reviewers in P and I categories. However, differences were reported in M and G classifications, where the software assigned more M ratings, and human reviewers assigned more G ratings [46]. Waade et al. (2021) examined agreement between radiographers and artificial intelligence (AI) software in assessing breast positioning. Agreement varied by criterion. Intraclass correlation coefficients between radiographers and AI ranged from κ = 0.06 to 0.92, depending on the positioning metric [47]. Agreement between radiographer pairs was higher, with a mean κ of 0.70, compared to a mean κ of 0.41 between radiographers and software [47]. Chan et al. (2022) presented a descriptive analysis of the capabilities of automated software tools. The report highlighted the software’s capacity to provide individualised feedback, benchmark performance across radiographers and facilities, and support training through review of performance trends over time [48].

## 4. Discussion

This review mapped the use of automated software tools for assessing mammographic image quality and radiographic technique, with a focus on radiographer-led acquisition in breast screening. Noted was the commercial availability of and publication on the technology from one vendor only. The evidence from the five included studies however points to a growing shift in practice where image quality assurance is increasingly supported by software tools such as Volpara Analytics and TruPGMI^TM^. These tools aim to address long-standing concerns about variability in image quality assessment technique, inconsistent feedback mechanisms, and the subjectivity of traditional visual grading systems used by radiographers and audit teams [8,20,31,35].

Across the reviewed studies, the primary application of automated tools was in improving consistency and reproducibility of image quality assessment. Visual grading systems like PGMI or EAR have well-documented limitations, including low inter-rater reliability and challenges in scaling across large programs [20]. The studies by Pickard et al. (2022) and Waade et al. (2021) highlight this issue. Both reported variability in how image quality and positioning were rated between human reviewers and software, particularly for criteria that are more nuanced or subjective, such as skin folds or nipple in profile [46,47]. However, the automated systems demonstrated high consistency in evaluating key parameters and flagged discrepancies and trends that may be otherwise overlooked in routine practice. This highlights the capacity of AI-based tools to function not just as quality monitors but also as a second layer of assurance that supports clinical governance in population screening programs.

Importantly, the adoption of these tools also appears to support radiographer learning and skill development. Chan et al. (2022) emphasized the educational value of these systems, noting their ability to provide individualised feedback, track performance trends over time, and benchmark outcomes across sites or against national standards [48]. This type of feedback is often missing from routine radiographic practice, where formal performance reviews can be infrequent or variable depending on workplace structure. Breast screening accreditation standards dictate the frequency of monitoring and feedback [30,32,33,36], however these vary between programs and are confounded in most cases by the subjectivity inherent in visual grading systems including PGMI IES. In this sense, automated systems may fill an important gap, not only by flagging deficiencies and benchmarking, but by promoting immediate reflective practice, accountability, and self-assessment among radiographers. This aligns with broader movements in health professions education toward formative feedback and continuous quality improvement (CQI) [49]. Technology adoption in radiography and healthcare to support CQI plays a significant role in achieving healthcare outcomes and enhancing quality of service and care. However, the integration of quality assurance tools in the health setting presents cultural, technical, structural and strategic barriers, with these challenges being amplified in low resource settings [49]. These are critical considerations for the real-world adoption of QA tools.

Several studies also suggest potential downstream benefits beyond individual performance. Gennaro et al. (2023) and Eby et al. (2023) demonstrated measurable improvements in technical quality and reductions in repeat or recall rates, which are directly relevant to patient safety, radiation exposure, and healthcare resource use. Reducing technical repeats not only minimises unnecessary radiation but may also lower patient anxiety and workflow interruptions, particularly in high-throughput screening settings [44,45]. Furthermore, consistent application of positioning and compression standards can help reduce missed lesions due to suboptimal image quality, although none of the included studies evaluated this explicitly. While these studies provide early signals of operational and clinical benefits, larger-scale evaluations are needed to confirm whether these tools influence longer-term outcomes such as cancer detection rates, diagnostic intervals, or false positives. This is a critical gap in the current research that would benefit from prospective studies that can validate the downstream clinical utility of these tools.

Despite these promising developments, several limitations were apparent across the included studies. Most notably, there was a lack of detail regarding the operational implementation of these tools. None of the studies addressed how radiographers were trained to interpret or use the feedback provided, whether the software altered workflow or consultation time, or how staff responded to the integration of automated review processes. As such, the feasibility and acceptability of widespread adoption remains uncertain. Software uptake may be influenced by perceptions of surveillance, perceived threat to professional autonomy, or a lack of clarity around thresholds for intervention.

Concerns around surveillance, autonomy and intervention may be considered to be less likely in the breast screening setting as unlike other areas of radiography, breast screening radiographers work under strict accreditation standards that include routine monitoring and reporting [30,32,33,36]. In Australia the BreastScreen Australia National Accreditation Standards (BSA NAS) include protocol 2.4 which includes the minimum requirement that requires that “*The Service and/or State Coordination Unit (SCU) demonstrates annually that each radiographer and mammography practitioner achieves 50% or greater P or G ratings in a PGMI evaluation of 50 randomly selected image sets*” [30]. Anecdotally some Services provide this feedback monthly to radiographers as a quality improvement initiative. Additionally, NAS 2.5.2 requires that “*The overall repeat rate for the Service and/or SCU to be ≤2% of all screening images*”, as a further means of assessing the quality of a Service [30]. The NHSPSP requires an audit of a minimum of 20 mammograms every 2 months [36]. Future studies should explore the behavioural and organisational dimensions of the implementation of automated image quality software, particularly in publicly funded screening programs where changes to practice require broad consultation and policy alignment.

Another gap in the evidence relates to cost and infrastructure. No study provided an economic evaluation or discussed the financial implications of adopting automated software. This includes licensing costs, interoperability with existing Picture Archiving and Communication Systems (PACS) and Reporting Information Systems (RIS), staff training or the information technology (IT) support required to sustain regular use. For many screening services, particularly those operating under budget constraints, these are critical factors in determining viability. While the clinical potential of these tools appears clear, their scalability may depend on demonstrating a return on investment through quality improvements, reduced recalls, improved breast cancer detection or better audit compliance.

In terms of generalisability, all five studies were conducted in high-resource settings. It remains unclear how automated evaluation tools would perform in low-resource or rural environments where radiographers may be less experienced or have limited access to continuing professional development. Indeed, many low-resource settings or those with less developed screening infrastructure have not been able to progress digital technology and are still using film screen acquisition systems, making image evaluation software integration redundant [50,51]. These settings often face higher rates of technical deficiency and workforce variability, making them ideal test cases for software-driven quality assurance where digital infrastructure is available. However, issues such as equipment compatibility, internet access, and local workflows must be considered. Broader evaluation in diverse settings is needed to confirm the equity and flexibility of these tools across different healthcare systems.

Lastly, the studies focused heavily on technical indicators of quality without examining clinical outcomes. For example, while Eby et al. (2023) reported reductions in technical recalls and improvements in positioning metrics, it is not known whether these improvements translated into enhanced diagnostic accuracy or patient experience. Similarly, although Gennaro et al. (2023) observed better compression performance, the impact on lesion visibility or cancer detection remains speculative [44,45]. Longitudinal studies linking software-generated metrics with screening outcomes would provide more meaningful evidence for policymakers and clinical leaders considering adoption.

The low yield of this study is not considered a limitation as even when few or no studies are identified, scoping reviews remain valuable for mapping gaps in the literature, informing evidence based practice, documenting the absence of evidence and guiding future research agendas [52,53,54].

In summary, this is the first scoping review to systematically investigate automated software used at the image acquisition stage in radiographer-led workflows. Although this review suggests that automated software tools hold significant promise for improving image quality assessment in mammographic screening the current evidence base is heavily centered on a single vendor (Volpara/TruPGMI^TM^), raising concerns about generalisability and vendor bias. Automated software tools appear to support radiographers in achieving technical excellence, offer structured feedback mechanisms, and promote consistency through benchmarking within and across services. While early evidence is encouraging, further research is needed to understand implementation barriers, cost-effectiveness, and clinical utility.

## Figures and Tables

**Table 1:** Search strategies for three databases.

Database	Searches		Results
PUBMED 1 March 2025	(“Mammography”[MeSH Terms] OR (“breast tomosynthes*s”[Title/Abstract] OR “mammograph*”[Title/Abstract] OR “tomosynthes*s screening”[Title/Abstract] OR “mammogram*”[Title/Abstract] OR “breast imag*”[Title/Abstract])) AND (“image quality”[Title/Abstract] OR “software evaluation”[Title/Abstract] OR “Volpara”[Title/Abstract] OR (“quality”[Title/Abstract] OR “artificial intelligence”[Title/Abstract] OR “technology assessment”[Title/Abstract])) AND ((“radiographer*”[Title/Abstract] OR “mammographer”[Title/Abstract] OR (“breast radiograph*”[Title/Abstract] OR “technologist*”[Title/Abstract])) NOT “radiologist”[Title/Abstract]) AND 2014/01/01:2025/12/31[Date-Publication]		84
EMCARE 14 March 2025	#1	mammography/	23,741
	#2	(breast tomosynthes?s or mammograph* or tomosynthes*s screening or mammogram* or breast imag*).mp.	29,726
	#3	1 or 2	29,726
	#4	(image quality or software evaluation or Volpara or quality or artificial intelligence or technology assessment).mp.	950,191
	#5	radiological technologist/	1481
	#6	(radiographer* or mammographer or breast radiograph* or technologist*).mp.	7462
	#7	5 or 6	7462
	#8	radiologist.mp.	40,166
	#9	7 not 8	6105
	#10	3 and 4 and 9	142
	#11	limit 10 to yr = “2014 -Current”	74
SCOPUS 14 March /2025	((TITLE-ABS-KEY (radiographer* OR mammographer OR “breast radiograph*” OR technologist*)) AND NOT (TITLE-ABS-KEY (radiologist))) AND (TITLE-ABS-KEY (“image quality” OR “software evaluation” OR volpara OR quality OR “artificial intelligence” OR “technology assessment”)) AND (TITLE-ABS-KEY (“breast tomosynthes*s” OR mammograph* OR “tomosynthes*s screening” OR mammogram* OR “breast imag*”)) AND PUBYEAR > 2013 AND PUBYEAR < 2026		107
	Citation Searching		3
Total			268
Duplicates			123
Total with duplicates removed			142

**Table 2:** Use of automated software evaluation to enhance radiographers’ performance in breast imaging.

Study, Year and Country	Study Design	Imaging Modality: 2D Full Field Digital Mammography (2D FFDM/Digital Breast Tomosynthesis (DBT)	Unit/Software/Tool Used	Sample Size	Radiographer Characteristics	Main Outcomes	Reported Limitations
Gennaro, G. et al., 2023; Italy [44]	Retrospective longitudinal analysis of prospective cohorts; observational; performed at one institution	2D FFDM/DBT	Hologic/Volpara Analytics/TruPGMI^TM^	One facility; six radiographers; 2407 women in the pre-software training cohort/ 3986 in the post-software cohort	Screening radiographers with 0–25 years experience;	Automated image quality analysis can improve the positioning and compression performance of radiographers, which may ultimately lead to improved screening outcomes.	Single centre and small number of radiographers; evaluation of short-term impact of software use on positioning and compression performance (only); included only women aged 46–47
Eby P. et al., 2023; United States [45]	Retrospective analysis of screening mammography image quality data	2D FFDM	Volpara Analytics/ TruPGMI^TM^	Nine facilities; 40 technologists 198,054 images and 42 technologists, 211,821 images	Screening radiographers	Rapid objective feedback on mammographic images is a major advantage to AI software analysis. Increases in all objectively measured IQ indicators following AI software implementation demonstrates the potential of AI software to improve IQ and reduce patient TR.	Inability to match individual studies due to the level of aggregation from the two data sources; it was not possible to assess the direct impact on patient outcomes; dose or to interrogate the specific predictors of technical recall (TR) or inadequate (I) images
Pickard, M et al., 2022; Belgium [46]	Retrospective analysis of screening mammography image quality	2D FFDM	Volpara TruPGMI^TM^	127 mammographic screening exams (MLO and CC views)	Screening radiographers	Automated image quality assessment software overcomes the issue of subjectivity and high reader variability.	Images acquired on mammography systems of one vendor; only one automatic evaluation of mammography positioning software is available and was tested; the number of readers was limited to two (one radiographer and one radiologist) discordant cases were managed y a second radiologist; the number of mammograms (*n* = 127) evaluated was also limited
Waade, GG. et al., 2021; Norway [47]	Randomised, retrospective analysis of screening mammography image quality	2D FFDM/DBT	GE Senographe Pristina 3 D Breast Tomosynthesis™/Volpara TruPGMI^TM^	Two hundred screening radiographers; 17,951 women, 14 breast centres.	Screening radiographers	AI has great potential in image quality and breast positioning assessment in mammographic screening by reducing subjectivity. However, there is varying agreement between radiographers and AI for several breast positioning criteria.	Limited criteria selected; criteria selected limited by available output from the AI system evaluated only assessed selected positioning criteria, no overall image quality or technical errors.
Chan, A. et al., 2022; Australia/New Zealand Book Chapter [48]	Book Chapter	Book Chapter	Volpara TruPGMI^TM^	Book Chapter	Book Chapter	Visual inspection of mammograms is subjective and time-consuming; consistent, objective, and ongoing feedback about breast positioning quality is challenging. Automated evaluation of breast positioning can: Allow individual review their of performance (overall and over time), as well as benchmark results against the facility and globally; Aid better understanding of performance, facilitate performance reviews and identify areas to target for training Support realistic objectives and goals based on benchmarking and individualised trends; Identify focus areas for improvement, by reviewing feedback down to the level of individual metrics; An automated approach to assessing positioning: Achieves and maintains a high standard of mammographic image quality; Provides an objective training program to advance positioning performance Facilitates external inspections and quality assurance programs	

## Data Availability

The original contributions presented in this study are included in the article. Further inquiries can be directed to the corresponding author.

## Abbreviations

The following abbreviations are used in this manuscript:

DBT	digital breast tomosynthesis
ACR	American College of Radiology
PGMI	Perfect (P), Good (G), Moderate (M) Inadequate (I)
IES	image evaluation system
NHSBSP	National Health Service Breast Screening Programme
UK	United Kingdom
CC	craniocaudal
MLO	mediolateral oblique
EAR	Excellent (E), Acceptable (A) or Repeatable (R)
PRISMA-ScR	Preferred Reporting Items for Systematic Reviews and Meta Analyses extension for Scoping Reviews
CAD	computor aided detection
MRI	magnetic resonance imaging
US	ultrasound
BSA NAS	BreastScreen Australia National Accreditation Standards
SCI	State Coordination Unit
PACS	Picture Archiving and Communication Systems
RIS	Reporting Information Systems
AI	artificial intelligence
IT	information technology
OSF	Open Science Framework
